# Resveratrol Butyrate Ester Protects Adenine-Treated Rats against Hypertension and Kidney Disease by Regulating the Gut–Kidney Axis

**DOI:** 10.3390/antiox11010083

**Published:** 2021-12-29

**Authors:** Chien-Ning Hsu, Chih-Yao Hou, Chi-I Chang, You-Lin Tain

**Affiliations:** 1 Department of Pharmacy, Kaohsiung Chang Gung Memorial Hospital, Kaohsiung 83301, Taiwan; cnhsu@cgmh.org.tw; 2 School of Pharmacy, Kaohsiung Medical University, Kaohsiung 80756, Taiwan; 3 Department of Seafood Science, National Kaohsiung University of Science and Technology, Kaohsiung 81157, Taiwan; chihyaohou@webmail.nkmu.edu.tw; 4 Department of Biological Science and Technology, National Pingtung University of Science and Technology, Pingtung 91201, Taiwan; 5 Department of Pediatrics, Kaohsiung Chang Gung Memorial Hospital and Chang Gung University College of Medicine, Kaohsiung 83301, Taiwan; 6 Institute for Translational Research in Biomedicine, Kaohsiung Chang Gung Memorial Hospital, Kaohsiung 83301, Taiwan

**Keywords:** chronic kidney disease, aryl hydrocarbon receptor, butyrate, developmental origins of health and disease (DOHaD), hypertension, gut microbiota, resveratrol, short chain fatty acid

## Abstract

Despite recent advances in pharma-nutritional management, chronic kidney disease (CKD) remains an increasingly prevalent disorder. Resveratrol, a pleiotropic phytochemical, has been found to reduce the risk for several chronic diseases. Considering the low bioavailability of resveratrol, we recently synthesized resveratrol butyrate ester (RBE) via the esterification of resveratrol with butyrate. The aim of this study was to examine the effectiveness of RBE as regards protection from hypertension and kidney damage and explore the underlying mechanisms using a young rat adenine-induced CKD model. Three-week-old male Sprague Dawley rats received regular or 0.5% adenine chow for three weeks. Three groups of adenine-fed CKD rats (N = 8/group) received resveratrol (50 mg/L), or a low dose (25 mg/L) or high dose (50 mg/L) of RBE in drinking water from week 6 to week 12. As compared with the controls, adenine-treated rats had markedly increased creatinine levels and blood pressure, which was associated with renal hypertrophy and decreased creatinine clearance. Treatment with resveratrol or a low or high dose of RBE, similarly protected adenine-fed rats against hypertension and kidney damage. CKD-induced hypertension is associated with an altered gut microbiota profile, dysregulated renal short chain fatty acid (SCFA) receptor expression, activation of the aryl hydrocarbon receptor (AhR) signaling pathway, and reduced nitric oxide bioavailability. We found gut microbiota compositions were shaped differentially by resveratrol and RBE treatment in adenine-treated CKD rats. The beneficial effect of high-dose RBE was associated with reduced renal expression of SCFA G protein-coupled receptor 41 (GPR41) and olfactory receptor 78 (Olfr78), antagonizing the AhR signaling pathway, and increased abundance of beneficial bacteria such as genera *Akkermansia*, *Blautia*, and *Enterococcus*. Our study provided the first evidence documenting RBE as a novel phytochemical supplement targeting the gut–kidney axis to protect against adenine-induced kidney damage and hypertension.

## 1. Introduction

Chronic kidney disease (CKD) is a growing global health issue that affects approximately 10% of the world’s population [1]. CKD can originate in infancy and childhood [2]. Critically, children with CKD are at increased risk of morbidity and mortality and are particularly vulnerable to complications associated with CKD [3]. Hypertension is the leading complication of pediatric CKD [4]. Prior research indicated that more than half of children with CKD display blood pressure (BP) abnormalities [4], even in the early stages of CKD [5]. Accordingly, early detection and management of hypertension in children with CKD is crucial to halt the growing global CKD epidemic.

Currently, there are several animal models of adult CKD, but too little attention has been paid to pediatric CKD models. Different degrees of CKD can be achieved by varying the concentration of adenine in the diet [6]. Our previous work showed that female adult Sprague Dawley rats treated with 0.5% adenine developed CKD [7]. The current effort aimed to evaluate the ability of young rats receiving 0.5% adenine treatment for three weeks to mimic human pediatric CKD.

Several mechanisms are involved in the development of CKD and hypertension, including oxidative stress, nitric oxide (NO) deficiency, aberrant renin-angiotensin system (RAS), and an imbalanced gut microbiome [8,9]. Emerging evidence links the gut microbiota and microbial metabolites with the gut-kidney axis in the development of hypertension and kidney disease [9]. Conversely, gut microbiota-targeting therapies have shown benefits against several chronic diseases, including hypertension [10].

Resveratrol is a pleiotropic phytochemical belonging to the stilbene family [11]. Population studies show that increased consumption of phytochemicals from fruits and vegetables is associated with a reduced risk of many chronic diseases [12]. Resveratrol exhibits wide-ranging beneficial effects, including antioxidant and anti-inflammation activity, prebiotic function, restoration of NO bioavailability, and antagonist activity on the aryl hydrocarbon receptor (AhR), etc. [13,14]. Our previous work indicated that resveratrol supplementation protected maternal CKD-induced hypertension in adult offspring coincided with alterations in the gut microbiota and short chain fatty acids (SCFAs), the main metabolites produced by the microbiota [15]. However, it is currently unknown whether resveratrol therapy can protect young adenine-treated CKD rats against hypertension and whether the mechanism is actually associated with the gut microbiota.

Notably, the low bioavailability of resveratrol reduces its efficacy and clinical translation [16]. We recently synthesized resveratrol butyrate ester (RBE) via the esterification of resveratrol with the SCFA butyrate [16]. We also demonstrated that RBE exhibits a higher antioxidant capacity than resveratrol itself [17]. Given that RBE has been shown to produce highly beneficial biological effects [18], the aim of the current study was: to (1) evaluate the effectiveness of RBE at low and high doses vs. resveratrol in the treatment of hypertension in a model of pediatric CKD; and (2) elucidate the mechanisms related to the beneficial effects, with a focus on the gut-kidney axis.

## 2. Materials and Methods

### 2.1. Animal Model

All animal procedures were performed in Kaohsiung Chang Gung Memorial Hospital with prior approval of the protocol by the Institutional Animal Care and Use Committee (approval number 2020101201). Sprague Dawley (SD) rats were obtained from BioLASCO Taiwan Co., Ltd., Taipei, Taiwan. The rats were maintained in our AAALAC-accredited animal facility, with a light-controlled environment (12-h light/12-h dark cycle; 22 °C) and a relative humidity of 55%. Chow and sterile tap water were available ad libitum. Mating was achieved by placing one male and one female in a cage overnight, and successful mating was confirmed by the presence of a vaginal plug. Considering that hypertension and kidney disease occur at an earlier age in males than females [18], only males were studied in the current study.

**Figure 1:**
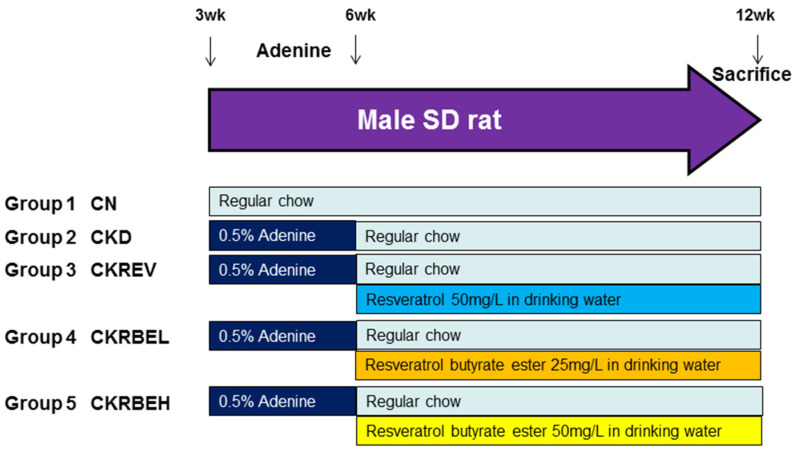
Experimental protocol used in the present study.

Three-week-old male rats received regular (CN group; N = 8) or 0.5% adenine chow (CKD group; N = 32) for 3 weeks. The dose of adenine was administered based on our previously established CKD model [7]. One group of adenine-fed CKD rats were treated with resveratrol (50 mg/L, CKREV group) in drinking water from week 6 to week 12. Resveratrol was prepared twice weekly by dissolving the drug in ethanol and then diluting it with water to a final concentration of 50 mg/L (10 mg/kg body weight/day), as described previously [19]. Water bottles were wrapped with aluminum foil to protect the solution from light. The other CKD rats received a low dose (25 mg/L, CKRBEL group) or a high dose (50 mg/L, CKRBEH group) of RBE in drinking water from week 6 to week 12. The dose of resveratrol used was based on a previously published paper [15]. The RBE was prepared based on our previously published work [17]. A total of five groups (N = 8/group) were analyzed: CN, CKD, CKREV, CKRBEL, and CKRBEH. The experimental design is illustrated in Figure 1.

We used the CODA BP system (Kent Scientific Corporation, Torrington, CT, USA) for the noninvasive determination of BP [7]. To ensure accuracy and reproducibility, the rats were acclimated to restraint and tail-cuff inflation for 1 week prior to the experiment. For each rat, five cycles were recorded at each time point. BPs were averaged from stable measures obtained from each rat. All rats were sacrificed at 12 weeks of age. Feces from each rat (N = 8/group) were collected the morning prior to sacrifice. Later, collected fecal samples were stored at −80 °C. Blood samples were collected into heparinized tubes. Kidneys were harvested after perfusion with phosphate buffered saline and stored at −80 °C. Creatinine was analyzed in both plasma and urine samples using a high-performance liquid chromatography (HPLC) method, as described previously [7].

### 2.2. Analysis of NO Pathway by HPLC

Plasma levels of l-arginine (the substrate for NO synthase), l-citrulline (the precursor of l-arginine), and asymmetric and symmetric dimethylarginine (ADMA and SDMA, both inhibitors of NO synthase) were determined according to our validated protocol [7]. We used a HPLC method (HP series 1100; Agilent Technologies Inc., Santa Clara, CA, USA) with fluorescence detection for OPA/3mercaptopropionic acid (3MPA) derivatives [7].

### 2.3. Analysis of Plasma SCFA Levels by GC-MS

We used an Agilent 7890B gas chromatograph (Agilent Technologies, Wilmington, NC, USA) equipped with an automated sampler to analyze plasma SCFA levels with our validated method [20]. These SCFAs included acetic acid, butyric acid, propionic acid, isovaleric acid, isobutyric acid, and valeric acid. Chromatographic separation was achieved using a DB-FFAP column (30 cm × 0.25 mm, 0.25 µm; Agilent Technologies, Wilmington, DE, USA). We used 2-ethylbutiric acid as the internal standard. The injection volume was 1 µL with a split ratio of 5:1 at 240 °C.

### 2.4. Quantitative Real-Time Polymerase Chain Reaction (qPCR)

RNA was extracted as described previously [16]. Two-step quantitative real-time PCR was performed using the iCycler iQ Real-Time PCR Detection System (Bio-Rad, Hercules, CA, USA) and Quantitect SYBR Green PCR Reagents kit (Qiagen, Valencia, CA, USA). A total of four SCFA receptors were analyzed, including olfactory receptor 78 (Oflr78), G protein-coupled receptor 41 (GPR41), GPR43, and GPR109A. We also determined AhR signaling pathway-related genes. These genes include AhR, AhR repressor (AHRR), 2,3,7,8-tetrachlorodibenzo-p-dioxin (TCDD)-inducible poly-ADP-ribose polymerase (TIPARP), AhR nuclear translocator (ARNT), and cytochrome P450 CYP1A1 (CYP1A1).

As the R18S expression level was constant across all the test samples, it was used as the reference gene for the internal control. Each sample was run in duplicate. For the relative quantification of gene expression, the comparative threshold cycle (Ct) method was employed. The fold-increase of the experimental sample, relative to the control, was calculated using Formula 2^−ΔΔCt^. Table 1 provides the PCR primer sequences.

### 2.5. Analysis of Gut Microbiota

As described previously [7], bacterial DNA from frozen stool specimens was extracted and analyzed by 16S rRNA metagenomic analysis on an Illumina Miseq platform (Illumina, San Diego, CA, USA) at the Biotools Co., Ltd. (Taipei, Taiwan). The sequences were processed using QIIME version 1.9.1. Sequences with a distance-based similarity of 97% or greater were clustered into operational taxonomic units (OTUs) using the USEARCH algorithm. The phylogenetic relationships were constructed based on a representative sequence alignment with Fast-Tree. We compared the patterns of α- and β-diversity of the microbial communities. Alpha diversity was analyzed using the ACE index. We assessed the β-diversity using analysis of similarities (ANOSIM) and partial least squares discriminant analysis (PLS-DA). We also used the linear discriminant analysis effect size (LEfSe) to identify high-dimensional biomarkers. The threshold of the linear discriminant was set to 3.

### 2.6. Statistical Analysis

All data are presented as mean ± the standard error of the mean (SEM). Comparisons within the five groups were first analyzed using one-way analysis of variance (ANOVA) followed by Tukey’s post hoc test. All statistical tests were considered statistically significant if *p* value < 0.05. Statistical analysis was performed in the Statistical Package for the Social Sciences software (SPSS Inc., Chicago, IL, USA).

## 3. Results

### 3.1. Blood Pressure and Renal Function

Table 2 shows death rates of zero in all groups. There was no difference in body weight between groups, while the kidney weight and kidney weight-to-BW ratios were lower in the CN group as compared to the other four groups.

**Figure 2:**
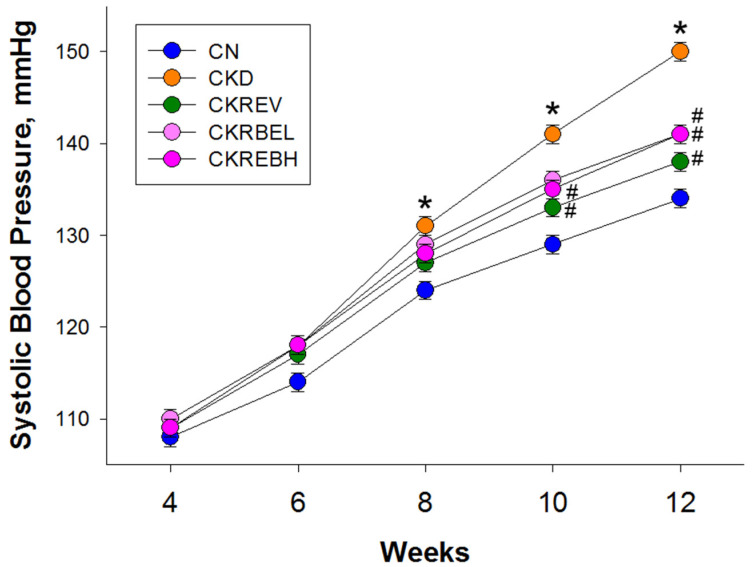
Effect of adenine-induced chronic kidney disease (CKD), resveratrol, or resveratrol butyrate ester on systolic blood pressure. N = 8/group; * *p* < 0.05 vs. CN; ^#^ *p* < 0.05 vs. CKD.

The values of systolic BP (SBP) from week 4 to 12 are depicted in Figure 2. We did not find differences in the baseline values of SBP. As compared to the CN group, adenine-induced CKD significantly increased SBP from 8 to 12 weeks of age. At week 10, the CKD-induced SBP increase was attenuated by resveratrol in the CKREV group and by high-dose RBE in the CKRBEH group. At 12 weeks of age, these CKD-induced increases in systolic and diastolic BPs and mean arterial pressures were prevented by resveratrol, and low-dose or high-dose RBE therapy (Table 2).

In addition, CKD rats exhibited a higher blood creatinine level and lower creatinine clearance rate than those in the ND rats. Creatinine clearance is a widely used test to estimate renal function, specifically the filtration rate of the glomeruli (GFR) in the kidneys. Adenine treatment caused an approximate 40% reduction in GFR. The reduction in renal function was similarly prevented by resveratrol, and low-dose and high-dose RBE therapy. Our results therefore indicate that changes in BPs and renal function were similarly improved by resveratrol and RBE therapy.

### 3.2. NO-Related Parameters

Results for the NO pathway are shown in Table 3. As compared to the ND group, the CKD group showed decreased plasma l-arginine levels and l-arginine-to-ADMA ratio. However, these changes were not significantly altered by resveratrol or RBE therapy. Collectively, NO deficiency developed in the CKD group, and thus, the protective effects of resveratrol and RBE are not likely related to the NO pathway.

### 3.3. Plasma SCFA Levels and Renal SCFA Receptors

Plasma SCFA levels are compared in Table 4. We found that the plasma SCFA levels were not different between the CN, CKD, and CKRBEH group. Table 4 demonstrates that plasma levels of propionic acid and butyric acid were lower in the CKREV and CKRBEL groups as compared to the CN group. Compared to the CKD group, resveratrol, and low-dose RBE therapy significantly reduced acetic acid. Additionally, resveratrol caused decreases in the plasma butyric acid level in the CKREV group as compared to the CKD group. Plasma levels of isobutyric acid, isovaleric acid, and valeric acid were not different among groups.

**Figure 3:**
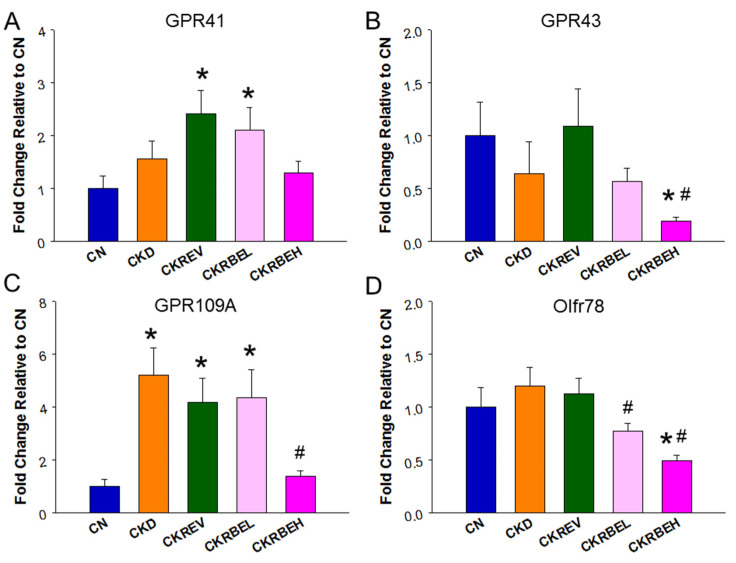
Effect of adenine-induced chronic kidney disease (CKD), resveratrol, or resveratrol butyrate ester on the renal SCFA receptors. The renal mRNA expression of (**A**) G protein-coupled receptor 41 (GPR41), (**B**) GPR43, (**C**) GPR109A, and (**D**) olfactory receptor 78 (Oflr78) were determined by qPCR. N = 8/group; * *p* < 0.05 vs. CN; ^#^ *p* < 0.05 vs. CKD.

Additionally, results of renal mRNA expression of the SCFA receptors are shown in Figure 3. Resveratrol and low-dose RBE were associated with increased GPR41 expression (Figure 3A). In contrast, high-dose BRE caused a reduction in GPR43 in the CKRBEH group in comparison with the CN and CKD groups (Figure 3B). Compared to controls, the renal GPR109A level was higher in the CKD, CKREV, and CKRBEL groups, and it was lower in the CKRBEH group (Figure 3C). Additionally, Figure 3D demonstrates that Olfr78 expression was reduced by low- and high-dose RBE as compared to the CKD group.

### 3.4. Aryl Hydrocarbon Receptor Signaling

**Figure 4:**
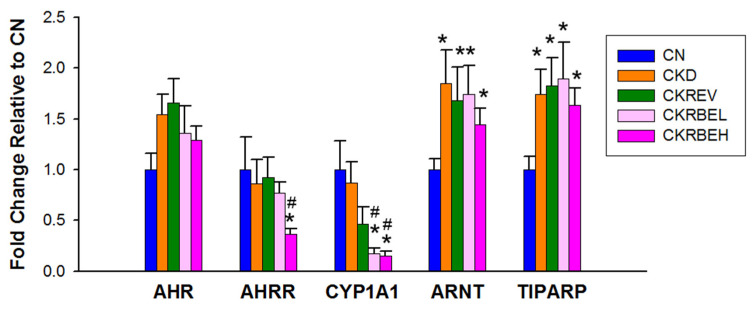
Effect of adenine-induced chronic kidney disease (CKD), resveratrol, or resveratrol butyrate ester on the mRNA expression of acryl hydrocarbon receptor (AhR) signaling pathway in the kidneys. N = 8/group; * *p* < 0.05 vs. CN; ^#^ *p* < 0.05 vs. CKD.

Renal mRNA expression of AhR signaling pathway-related genes are compared in Figure 4. There was no difference in AhR expression among the five groups. Among AhR-related genes, high-dose RBE therapy was associated with decreased AHRR and CYP1A1, but increased ARNT and TIPARP. Similarly, low-dose RBE therapy reduced CYP1A1 and increased ARNT and TIPARP expression. Compared to the controls, the CKD group showed increased ARNT and TIPART. However, resveratrol and RBE treatment had a negligible effect on their expression as compared to the CKD group.

### 3.5. Gut Microbiota Compositions

**Figure 5:**
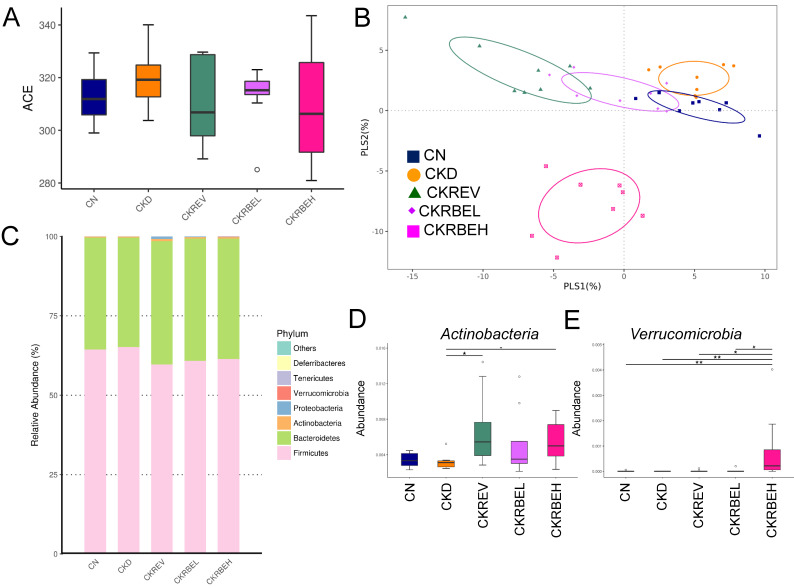
Effect of adenine-induced chronic kidney disease (CKD), resveratrol, or resveratrol butyrate ester on the gut microbiome. (**A**) α-Diversity measured by the abundance-based coverage estimator (ACE) index. (**B**) β-Diversity using the partial least squares discriminant analysis (PLS-DA). (**C**) Relative abundance of the top five phyla of the gut microbiota. Relative abundance of the phyla (**D**) *Actinobacteria* and (**E**) *Verrucomicrobia*. N = 8/group; * *p* < 0.05; ** *p* < 0.01.

We further determined α- and β-diversity metrics to assess the effects of resveratrol or resveratrol butyrate ester on the total gut microbial community. The microbial ACE index, i.e., the α-diversity metric, was not significantly different between groups (Figure 5A). To measure similarities between the microbial communities, two β-diversity metrics, i.e., the PLS-DA and ANOSIM, were examined. Scatterplots of the PLS-DA analysis are depicted in Figure 5B. The PLS-DA analysis revealed significant clustering according to the study group, indicating that the microbial community was distinctly altered by the CKD and RBE treatments. The ANOSIM analysis confirmed a significant difference between the CN group and the other groups (All *p* < 0.05), excepting the CKD group (*p* = 0.117). ANOSIM also showed that the difference between CKD and CKREV group was significant (*p* = 0.044). However, the difference between the CKD and CKRBEL group did not reach the significance (*p* = 0.22).

Both the controls and the CKD rats had typical microbiome structures. As shown in Figure 5C, most bacteria fell into the phyla *Firmicutes*, *Bacteroidetes*, *Proteobacteria*, *Actinobacteria*, and *Verrucomicrobia*. Although these bacterial phyla were detected in all groups, some of the phyla showed differences between the groups. Figure 5D illustrates that the abundance of the phylum *Actinobacteria* was increased in the CKREV and CKRBEH groups as compared to the CN group. The relative abundance of the phylum *Verrucomicrobia* was highest in the CKRBEH group (Figure 5E).

**Figure 6:**
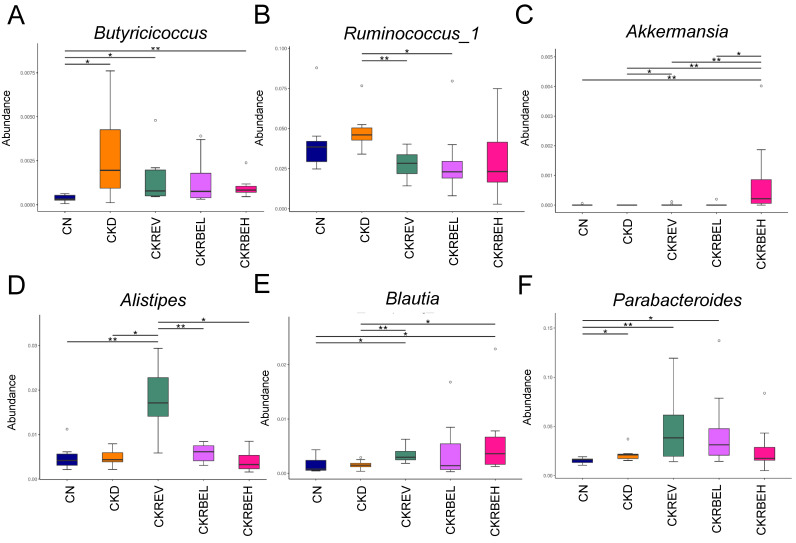
Effect of adenine-induced chronic kidney disease (CKD), resveratrol, or resveratrol butyrate ester on the gut microbiome. Relative abundance of the genera (**A**) *Butyricicoccus*, (**B**) *Ruminococcus_1*, (**C**) *Akkermansia*, (**D**) *Alistipes*, (**E**) *Blautia*, and (**F**) *Parabacteroides*. N = 8/group. * *p* < 0.05; ** *p* < 0.01.

At the genus level, the abundance of *Butyricicoccus* was significantly increased by CKD, which was prevented by resveratrol or high-dose RBE treatments (Figure 6A). Additionally, the abundance of *Ruminococcus_1* was higher in the CKD group in comparison with the CKREV and CKRBEL groups (Figure 6B). As a result, the CKRBEH group had the highest proportion of *Akkermansia* among the five groups (Figure 6C). Resveratrol treatment caused a significant increase in the genus *Alistipes* in the CKREV group as compared to the other groups (Figure 6D). Furthermore, the abundance of *Blautia* was lower in the CKD group as compared to the CKREV and CKRBEH groups (Figure 6E). Compared to the CN group, the abundance of the genus *Parabacteroides* was higher in the CKD, CKREV, and CKRBEL groups (Figure 6F).

**Figure 7:**
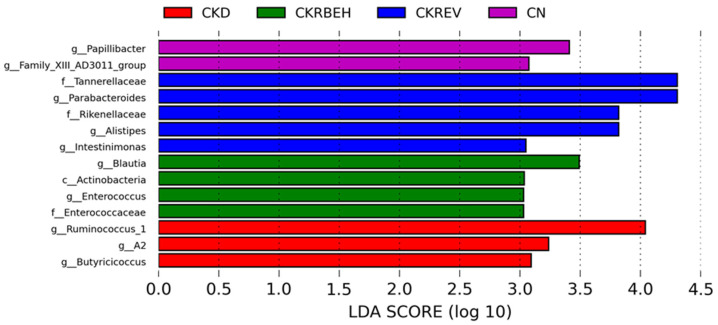
Linear discriminant analysis effect size (LEfSe) was assessed for biomarker discovery. The most enriched bacterial taxa in the CKD (red), CKRBEH (green), CKREV (blue), and CN groups (purple) are shown.

To identify taxonomic differences between groups, we used the LEfSe tool with a relatively stringent LDA value of 3 (Figure 7). The LEfSe analysis revealed a high abundance of the genera *Butyricicoccus* and *Ruminococcus_1* in the CKD group. Resveratrol treatment resulted in a higher abundance of the genera *Parabacteroides*, *Alistipes*, and *Intestinimonas* in the CKREV group. In particular, the genera *Blautia* and *Enterococcus* were distinguished by LEfSe as having a high LDA score in the CKRBEH group.

## 4. Discussion

Our study demonstrates that low-dose RBE (25 mg/L) is as effective as resveratrol (50 mg/L) in protecting from hypertension and deterioration in renal function in a pediatric CKD rat model. The important findings from our data are as follows: (1) similar to adults, adenine-fed young rats develop major characteristics mimicking human CKD; (2) both resveratrol and RBE treatments protected adenine-treated rats against hypertension and renal dysfunction; however, the protective effect of RBE was not dose dependent; (3) CKD-induced hypertension coincided with NO deficiency, increased renal GPR109A, ARNT and TIPART expression, and increased the abundance of the genera *Butyricicoccus* and *Ruminococcus_1*; (4) resveratrol and RBE treatment differentially shaped distinct gut microbiota profiles in adenine-treated rats; (5) the protective effect of resveratrol against hypertension accompanied alterations in several SCFAs-producing microbes, including *Alistipes*, *Blautia*, and *Parabacteroides*; and (6) high-dose RBE significantly reduced renal GPR43 and Olfr78 expression, decreased AhR-relating AHRR and CYP1A1, and increased the abundance of the genera *Akkermansia*, *Blautia*, and *Enterococcus*.

In support of prior research in adult rats [6,7], treatment of young rats with adenine induces CKD. In the present study, we developed a pediatric adenine-induced CKD model using adult rats, which is characterized by a ~40% GFR decline, renal hypertrophy, and hypertension. The renal protective effects of resveratrol in the presence of hypertension are in accordance with findings in both human and experimental studies [12,13,14] and with our previous report on a maternal CKD model [15]. We previously demonstrated that RBE can inhibit lipid accumulation and protect against liver damage in a bisphenol A exposure model [21,22]. To the best of our knowledge, this is the first study to demonstrate that RBE protects CKD rats against hypertension and renal dysfunction.

Low-dose RBE treatment (25 mg/L) and resveratrol were revealed to produce similar beneficial effects against hypertension and kidney damage in adenine-induced CKD rats. However, no difference was observed between the low- and high-dose groups, suggesting that the protective effects of RBE are not dose dependent. As compared with the bioavailability of resveratrol, our prior work revealed that RBE significantly suppresses the associated injuries at a lower dose [21]. Our previous study demonstrated that RBE was a mixture of pristine resveratrol (~26.6%) and five ester derivatives (~73.4%) [21,23]. Resveratrol can be rapidly metabolized in our body to produce different metabolites such as sulfate and glucuronide conjugate metabolites, piceatannol, and dihydroresveratrol in the circulation and target organs [24,25]. Although we identified and separated five esterified derivatives of RBE, their bioavailability and metabolism require further research. As the protective actions of each RBE ester derivative may be differential, there is a need to explore their differences for future practical applications of RBE complexes.

Several mechanisms contribute to the pathogenesis of CKD-induced hypertension. Our study provides further evidence that supports the close link between CKD-induced hypertension and aberrant AhR activation, alterations in gut microbiota compositions, and reduced NO bioavailability.

Although CKD had a negligible effect on plasma SCFA levels, the beneficial effect of resveratrol and RBE against CKD-induced hypertension might involve its ability to mediate SCFA receptors. SCFAs can regulate BP by stimulating their receptors, including GPR 41 and GPR109A, to reduce BP and can be counteracted through GPR43 and Olfr78 to induce vasoconstriction [26]. Accordingly, resveratrol-enhanced GPR 41 and GPR109A expression may have a vasodilatory effect to improve high BP in CKD rats. Moreover, high-dose RBE protection against hypertension is related to low GPR43 and Olfr78 expression, shifting the balance between vasodilation and vasoconstriction towards vasodilation.

Another of RBE’s protective mechanisms against CKD-induced hypertension may be associated with the mediation of the AhR signaling pathway. We previously reported that exogenous AhR ligand TCDD-induced hypertension coincided with the activation of the AhR signaling pathway [27]. This is supported by the present observations, which demonstrated that CKD-induced hypertension coincides with the activation of the AhR signaling pathway as represented by increased ARNT and TIPARP expression. Considering that the activation of the AhR/CYP1A1 axis induces vasoconstriction [27], high-dose RBE inhibition of renal CYP1A1 expression might, at least in part, contribute to its protective effects in BP control.

Prior research shows that the beneficial effects of resveratrol are also relevant to its ability to alter the gut microbiota with a prebiotic effect [14,28]. Although resveratrol treatment shaped a different microbiome composition as compared to the CKD group, there was no difference in α-diversity between the two groups. Interestingly, the data in this work showed that resveratrol protects against adenine-induced hypertension and kidney damage, which coincides with an abundance of the genera *Parabacteroides*, *Blautia*, and *Alistipes.* Given that the abundance of the genera *Alistipes* and *Blautia* was inversely associated with BP [29,30], that resveratrol increased the abundance of the genera *Parabacteroides*, and *Alistipes* was associated with protection against kidney damage in a diabetic nephropathy model [31], it is possible that resveratrol can influence these microbes towards renoprotection and have a BP-lowering benefit.

According to our data, high-dose RBE increased the abundance of the phylum *Verrucomicrobia*, i.e., the genera *Akkermansia*, *Blautia*, and *Enterococcus*. It is now believed that *Akkermansia*, a genus in the phylum *Verrucomicrobia*, acts as a beneficial microbe in the gut [32]. Similarly, *Blautia* and *Enterococcus* have been shown to exhibit probiotic properties [33,34].

Additionally, we observed that resveratrol or RBE caused a reduction in the butyric acid level and butyrate-producing bacteria such as *Butyricicoccus* and *Ruminococcus_1*. Our results contradict those previously published that report that decreases in butyrate-producing bacteria were accompanied with hypertension [9]. It is possible that the decreases caused by resveratrol or RBE are a negative feedback compensatory response to their BP-lowering effect.

Furthermore, our data are consistent with prior research demonstrating that adenine-induced hypertension and kidney dysfunction are associated with NO deficiency [7], as represented by decreased l-arginine-to-ADMA ratio. However, resveratrol and RBE had no effects on NO bioavailability in this study.

Our study has certain limitations. First, we are well aware that two testing doses of RBE in the present study might not completely compare its effectiveness with resveratrol. Additional studies with multiple test doses are warranted. Second, the present study only tested young male rats. Whether sex differences exist in the therapeutic response of resveratrol or RBE requires further evaluation. Since RBE is also involved in protecting against oxidative stress, additional studies are needed to elucidate whether these protective effects also play a role in the renoprotection of CKD-induced hypertension. Third, we restricted resveratrol treatment to the CKD group because we and others have shown resveratrol has no effect on BP in normotensive controls [35,36]. However, additional studies are needed to investigate whether RBE affects the intestinal microbiota in normal controls in a dose-dependent manner. Fourth, extensive experiments, including in vitro and in vivo studies, are required for a thorough examination of the mechanistic links involved in the protective effects of RBE, which may include oxidative stress and inflammation. Moreover, we did not analyze renal histopathology for two reasons: first, we measured GFR using creatinine clearance to represent renal function, and this parameter is suitable to represent the severity of kidney damage; second, our previous study demonstrated that the severity of glomerular and tubulointerstitial injuries are related to the reduction in renal function in this adenine-induced CKD model [7]. However, attention needs to be paid to the potential impact that RBE has on the histopathology of kidney damage. Finally, the anti-hypertensive effects of resveratrol and RBE may be attributed to other organs that regulate BP. Additional research is required to evaluate the BP-lowering effect on other BP-controlled organs.

## 5. Conclusions

Conclusively, this study provides the first evidence that resveratrol butyrate ester is a potential therapy targeting the gut-kidney axis to protect against adenine-induced kidney damage and hypertension. Although our study revealed novel mechanistic aspects related to resveratrol butyrate ester, these results require further investigation and clinical translation.

## Figures and Tables

**Table 1:** PCR primer sequences.

Gene	Forward	Reverse
*GPR41*	5 tcttcaccaccgtctatctcac 3	5 cacaagtcctgccaccctc 3
*GPR43*	5 ctgcctgggatcgtctgtg 3	5 cataccctcggccttctgg 3
*GPR109A*	5 cggtggtctactatttctcc 3	5 cccctggaatacttctgatt 3
*Olfr78*	5 gaggaagctcacttttggtttgg 3	5 cagcttcaatgtccttgtcacag 3
*AhR*	5 gtcctcagcaggaacgaaag 3	5 ccagggaagtccaactgtgt 3
*AHRR*	5 cagcaacatggcttctttca 3	5 tgaagcactgcattccagac 3
*CYP1A1*	5 gcactctggacaaacacctg 3	5 atatccaccttctcgcctgg 3
*ARNT*	5 gtctccctcccagatgatga 3	5 gctggtagccaacagtagcc 3
*TIPARP*	5 gttgagggccaattaccaga 3	5 gctcctggcacataatccat 3
*R18S*	5 gccgcggtaattccagctcca 3	5 cccgcccgctcccaagatc 3

*GPR41* = G protein-coupled receptor 41; *GPR43* = G protein-coupled receptor 43; *GPR91* = G protein-coupled receptor 91; Oflr78 = olfactory receptor 78; *AhR* = aryl hydrocarbon receptor; *ARNT* = Aryl hydrocarbon receptor nuclear translocator; *AHRR* = Aryl hydrocarbon receptor repressor; *TIPARP* = TCDD-inducible poly-ADP-ribose polymerase; *CYP1A1* = cytochrome P450 CYP 1A1; *R18S* = 18S ribosomal RNA.

**Table 2:** Weight, blood pressures, and renal function.

Groups	CN	CKD	CKREV	CKRBEL	CKRBEH
Mortality	0%	0%	0%	0%	0%
Body weight (BW) (g)	429 ± 21	430 ± 32	413 ± 28	426 ± 25	413 ± 37
Left kidney weight (g)	1.88 ± 0.14	3.18 ± 0.5 *	3.02 ± 0.35 *	2.99 ± 0.53 *	2.94 ± 0.43 *
Left kidney weight/100 g BW	0.44 ± 0.03	0.74 ± 0.07 *	0.73 ± 0.11 *	0.70 ± 0.11 *	0.71 ± 0.10 *
Systolic BP (mmHg)	134 ± 3	150 ± 4 *	138 ± 3 ^#^	141 ± 3 ^#^	141 ± 5 ^#^
Diastolic BP (mmHg)	92 ± 7	102 ± 5 *	92 ± 5 ^#^	89 ± 11 ^#^	92 ± 7 ^#^
Mean arterial pressure (mmHg)	106 ± 5	118 ± 4 *	108 ± 3 ^#^	107 ± 8 ^#^	108 ± 5 ^#^
Creatinine (μM)	31.6 ± 4.1	51.3 ± 6.4 *	35.5 ± 1.6 ^#^	33.2 ± 2.1 ^#^	33.5 ± 3.0 ^#^
Creatinine clearance rate (mL/min)	2.4 ± 0.3	1.4 ± 0.1 *	2.3 ± 0.3 ^#^	2.1 ± 0.2 ^#^	2.1 ± 0.3 ^#^

>N = 8/group; * *p* < 0.05 vs. CN; ^#^ *p* < 0.05 vs. CKD.

**Table 3:** Plasma levels of NO-related parameters.

Groups	CN	CKD	CKREV	CKRBEL	CKRBEH
l-citrulline (μM)	106.5 ± 9.6	120.7 ± 7.6	77.3 ± 7.1 *^#^	79.2 ± 4.5 *^#^	80.3 ± 6.5 *^#^
l-arginine (μM)	407.2 ± 33.9	256.4 ± 13.2 *	234.2 ± 13.9 *	243.5 ± 12.4 *	229.3 ± 21 *
Asymmetric dimethylarginine (μM)	1.88 ± 0.30	2.61 ± 0.26	2.41 ± 0.14	2.46 ± 0.26	1.91 ± 0.27
Symmetric dimethylarginine (μM)	1.38 ± 0.14	1.37 ± 0.08	1.38 ± 0.15	1.53 ± 0.08	1.33 ± 0.11
l-arginine-to-ADMA ratio (μM/μM)	252 ± 39	108 ± 16 *	103 ± 14 *	112 ± 17 *	139 ± 27 *

N = 8/group; * *p* < 0.05 vs. CN; ^#^ *p* < 0.05 vs. CKD.

**Table 4:** Plasma short chain fatty acid levels.

Groups	CN	CKD	CKREV	CKRBEL	CKRBEH
Acetic acid (μM)	525 ± 61	547 ± 55	391 ± 38 ^#^	371 ± 31 *^#^	412 ± 42
Propionic acid (μM)	2.74 ± 0.62	1.38 ± 0.33	0.92 ± 0.1 *	0.94 ± 0.19 *	1.38 ± 0.59
Isobutyric acid (μM)	2.5 ± 0.25	2.65 ± 0.54	2.19 ± 0.13	2.05 ± 0.19	2.33 ± 0.15
Butyric acid (μM)	3.55 ± 0.35	2.85 ± 0.32	1.98 ± 0.15 *^#^	2.53 ± 0.26 *	3 ± 0.44
Isovaleric acid (μM)	3.15 ± 0.76	2.56 ± 0.49	1.94 ± 0.63	1.74 ± 0.58	1.88 ± 0.46
Valeric acid (μM)	3.83 ± 0.86	4.96 ± 0.94	4.28 ± 0.54	4.12 ± 0.45	3.92 ± 0.58

N = 8/group; * *p* < 0.05 vs. CN; ^#^ *p* < 0.05 vs. CKD.

## Data Availability

Data is contained within the article.
